# IL-15 reprogramming compensates for NK cell mitochondrial dysfunction in HIV-1 infection

**DOI:** 10.1172/jci.insight.173099

**Published:** 2024-02-22

**Authors:** Elia Moreno-Cubero, Aljawharah Alrubayyi, Stefan Balint, Ane Ogbe, Upkar S. Gill, Rebecca Matthews, Sabine Kinloch, Fiona Burns, Sarah L. Rowland-Jones, Persephone Borrow, Anna Schurich, Michael Dustin, Dimitra Peppa

**Affiliations:** 1 Nuffield Department of Clinical Medicine and; 2 Kennedy Institute of Rheumatology, University of Oxford, Oxford, United Kingdom.; 3 Department of Hepatology, Centre for Immunobiology, Blizard Institute, Barts and the London School of Medicine and Dentistry, Queen Mary University of London, London, United Kingdom.; 4 Institute for Global Health UCL, London, United Kingdom.; 5 Royal Free London NHS Foundation Trust, London, United Kingdom.; 6 School of Immunology and Microbial Sciences, King’s College London, London, United Kingdom.; 7 Division of Infection and Immunity, University College London, London, United Kingdom.; 8 Mortimer Market Centre, Department of HIV, CNWL NHS Trust, London, United Kingdom.

**Keywords:** AIDS/HIV, Immunology, Mitochondria, NK cells

## Abstract

Dynamic regulation of cellular metabolism is important for maintaining homeostasis and can directly influence immune cell function and differentiation, including NK cell responses. Persistent HIV-1 infection leads to a state of chronic immune activation, NK cell subset redistribution, and progressive NK cell dysregulation. In this study, we examined the metabolic processes that characterize NK cell subsets in HIV-1 infection, including adaptive NK cell subpopulations expressing the activating receptor NKG2C, which expand during chronic infection. These adaptive NK cells exhibit an enhanced metabolic profile in HIV-1^–^ individuals infected with human cytomegalovirus (HCMV). However, the bioenergetic advantage of adaptive CD57^+^NKG2C^+^ NK cells is diminished during chronic HIV-1 infection, where NK cells uniformly display reduced oxidative phosphorylation (OXPHOS). Defective OXPHOS was accompanied by increased mitochondrial depolarization, structural alterations, and increased DRP-1 levels promoting fission, suggesting that mitochondrial defects are restricting the metabolic plasticity of NK cell subsets in HIV-1 infection. The metabolic requirement for the NK cell response to receptor stimulation was alleviated upon IL-15 pretreatment, which enhanced mammalian target of rapamycin complex 1 (mTORC1) activity. IL-15 priming enhanced NK cell functionality to anti-CD16 stimulation in HIV-1 infection, representing an effective strategy for pharmacologically boosting NK cell responses.

## Abstract 2

<p>Natural killer&nbsp;cells in HIV-1 infection exhibit metabolic defects, including mitochondrial dysfunction and structural alterations,&nbsp;that affect&nbsp;both canonical and adaptive subsets.</p>

## Graphical abstract

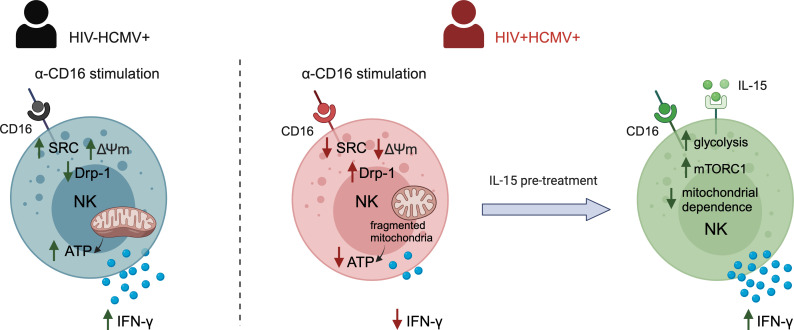

## Introduction

NK cells play a key role in antiviral immunity, and a number of studies implicate NK cells as critical contributors to the immune control of HIV-1 (1–3). It is well documented that persistent HIV-1 infection alters the subset distribution and functional capacity of NK cells (4–6). In particular, human cytomegalovirus (HCMV) coinfection, a hallmark of HIV-1–infected cohorts, triggers a dramatic expansion of differentiated NK cells expressing the activating receptor NKG2C, which prototypically characterizes adaptive NK cells (7–9). These expanded NKG2C^+^ NK cells coexpress CD57, a marker of maturation, and are influenced by human leukocyte antigen-E (HLA-E) presented peptides (10, 11). Notably, they resemble memory CD8 T cells and are imbued with enhanced capacity for antibody-dependent cellular cytotoxicity (ADCC) — in particular, increased IFN-γ production attributed to epigenetic remodeling of the *IFNG* locus (12, 13). While these attributes are considered important in the functional specialization of these NK cells, the development of these features under conditions of continuous stimulation/persistent inflammation during HIV-1 infection could lead to the establishment of functionally and metabolically exhausted NK cells akin to exhausted CD8 T cells. In keeping with this notion, chronic stimulation of adaptive NK cells through NKG2C ligation was recently found to lead to a molecular program of exhaustion that is shared between NK cells and CD8 T cells (14). Exhausted CD8 T cells are characterized by a number of metabolic defects. We and others have recently described how dysfunctional mitochondrial metabolism and reduced metabolic plasticity can lead to defective antiviral T cell function in HIV-1 infection (15, 16). However, to date, it remains unexplored how persistent HIV-1 infection contributes to the metabolic remodeling of NK cell subsets. Specifically, NK cell exhaustion could influence responses to CD16 engagement linked to vaccine-induced protective immunity against HIV-1 infection and phenotypes of viral control (17).

Metabolic regulation of T cell function is well characterized, and evidence of the importance of immunometabolism in facilitating robust NK cell functions is gradually emerging, too. Notably, impaired NK cell cellular metabolism has been implicated in obesity and cancer, providing a new framework for understanding NK cell functionality (18, 19). Studies from both human and murine models have demonstrated that NK cells activated through cytokine stimulation exhibit substantial increases in the rates of both glycolysis and oxidative phosphorylation (OXPHOS) pathways (20–23). However, the metabolic requirement of NK cells for optimal effector function, in terms of IFN-γ production, depends on the specific activation stimuli (24). In particular, receptor-mediated activation through anti-NK1.1 and anti-Ly49D in mice appears to require OXPHOS as an essential second signal for IFN-γ production and appears more susceptible to metabolic inhibition compared with cytokine stimulation (25).

Different metabolic programs are adopted by different NK cell subsets, and recent evidence suggests that distinct metabolic fingerprints underline the fate of NK cells (26, 27). Along these lines, adaptive NK cells emerging in response to HCMV infection in HIV-1^–^ adults have been reported to engage different metabolic pathways and exhibit enhanced mitochondrial fitness relative to their canonical counterparts (28). These adaptive CD57^+^NKG2C^+^ NK cell subpopulations display a superior respiratory capacity supported by increased mitochondrial mass, mitochondrial membrane potential, and upregulation of genes involved in the electron transport chain (ETC). The enhanced mitochondrial metabolism in adaptive NK cell subsets was found to be regulated by the chromatin-modifying transcriptional regulator AT-rich interactive domain-containing protein 5B (ARID5B) (28). These features may explain the increased functionality of adaptive NK cell subsets in HCMV-seropositive HIV-1^–^ donors, especially augmented IFN-γ production after CD16-dependent target recognition. The latter has been linked to increased activity of the mammalian target of rapamycin complex 1 (mTORC1) pathway, a well-established regulator of multiple metabolic processes (12). However, knowledge of NK cell metabolism in other chronic viral infections such as HIV-1, where HCMV coinfection is almost universal and plays a defining role in shaping the NK cell pool, remains in its infancy.

In this study, we sought to address the metabolic features of NK cell subsets during chronic HIV-1/HCMV coinfection. Our findings show that inefficient OXPHOS and loss of mitochondrial integrity in NK cells during HIV-1 infection influence responses to activating CD16 receptor stimulation. Such defects could be overcome in vitro by IL-15 metabolic reprogramming, which reduced the dependence of the response on receptor-mediated metabolic remodeling.

## Results

### NK cells from treatment naive HIV-1^+^ patients exhibit reduced oxidative metabolism.

**Figure 1:**
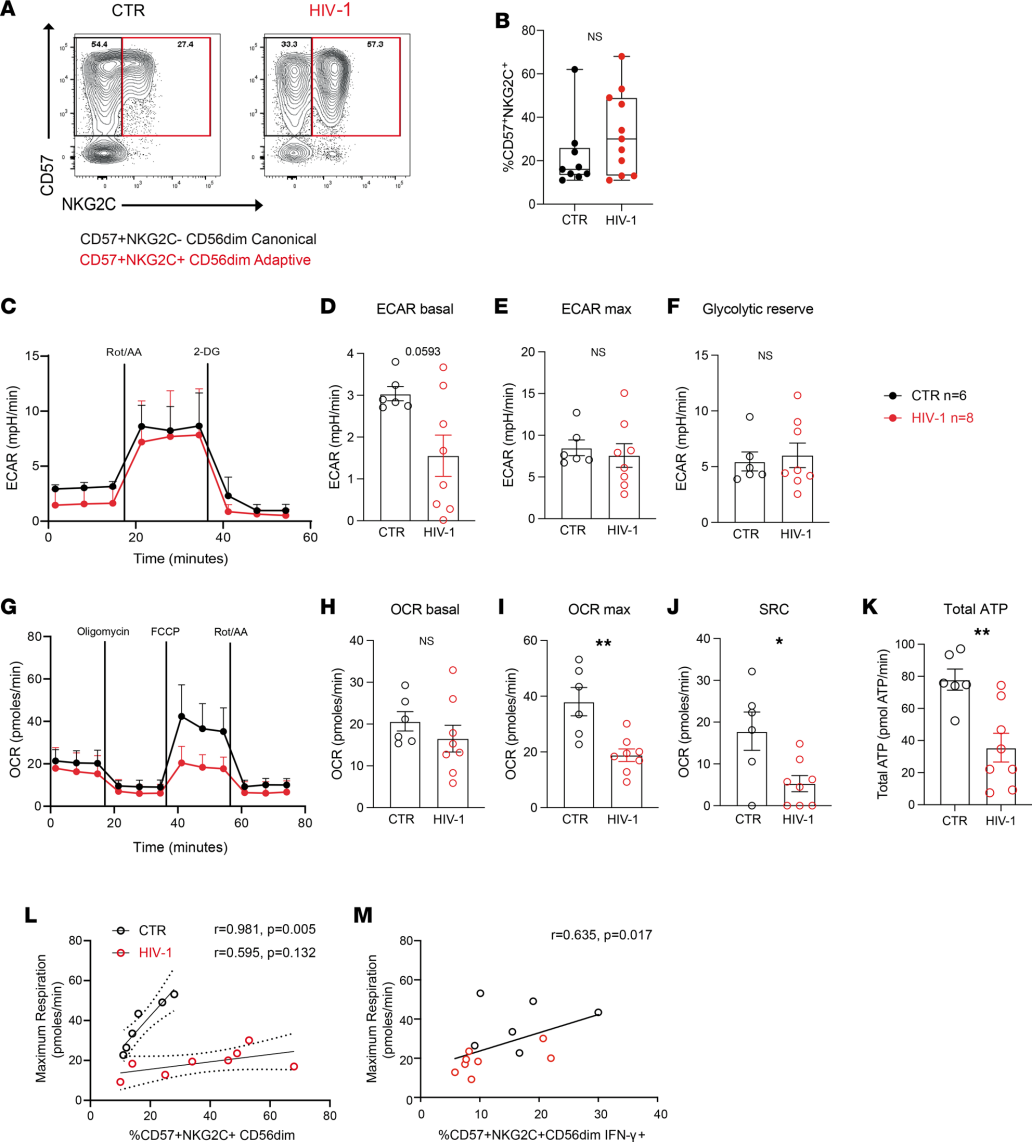
Analysis of the basal metabolic profile of NK cells in control (CTR) and HIV-1^+^ donors. (**A**) Representative example of flow cytometry plots showing canonical (NKG2C^–^CD57^+^) and adaptive (NKG2C^+^CD57^+^) NK cell subsets. (**B**) Summary data showing the frequencies of adaptive NK cells (CD56^dim^CD57^+^NKG2C^+^) in HIV-1^–^ (CTR) and HIV-1^+^ (HIV) donors. (**C**) Real-time analysis of aerobic glycolysis (determined from the extracellular acidification rate [ECAR]) in purified NK cells. (**D**–**F**) basal ECAR, maximal ECAR, and glycolytic reserve in isolated NK cells from *n* = 6 HCMV^+^ HIV-1^–^ CTR and *n* = 8 patients positive for HIV-1. (**G**) Real-time analysis of oxygen consumption rate (OCR) in isolated NK cells from both study groups. (**H**–**J**) Basal OCR, maximal OCR (OCR max), and spare respiratory capacity (SRC). Data are shown as mean ± SEM. (**K**) Summary of the total rate of ATP production by NK cells in CTR and HIV-1^+^ donors. (**L** and **M**) Correlation between maximal OCR and ex vivo percentage of adaptive CD57^+^NKG2C^+^CD56^dim^ NK cells (**L**) and proportion of CD57^+^NKG2C^+^CD56^dim^IFN-γ^+^ cells after CD16 triggering (**M**). The nonparametric Spearman test was used for correlation analysis. Sample triplicates were used for Seahorse assays. Significance determined by 2-tailed Mann-Whitney *U* test; *P < 0.05, **P < 0.01.

To study the metabolic profile of NK cells in HIV-1 infection, accounting for the influence of HCMV coinfection, we isolated total NK cells from HCMV seropositive treatment-naive HIV-1^+^ donors and HCMV-seropositive HIV-1^–^ donors (referred to as controls). All donors in this study were screened for the presence of adaptive CD57^+^NKG2C^+^ NK cells (Figure 1, A and B) as previously described (29). An extracellular flux analyzer was utilized to analyze the metabolic requirements of total NK cells (Supplemental Figure 1A; supplemental material available online with this article; https://doi.org/10.1172/jci.insight.173099DS1). The glycolytic rate assay was used to estimate the extracellular acidification rate (ECAR), a measure of lactate production and glycolysis at the basal state and after the addition of rotenone and antimycin A, which interfere with complex I and complex III of the ETC, respectively, and 2-deoxy-glucose (2-DG) a competitive inhibitor of glycolysis. Consistent with previous reports, the baseline activity of NK cells was low in both groups, and a nonsignificant trend toward lower basal ECAR was noted in the HIV-1^+^ group (Figure 1, C and D).

No significant differences in the rates of total NK cell maximal glycolysis and the glycolytic reserve were detected between the 2 groups, indicating that there were no major modulations of glycolytic pathways at baseline (Figure 1, E and F). Next, we assessed mitochondrial respiration by measuring oxygen consumption rates (OCR), a measure of OXPHOS, at basal levels and following the addition of a stressor mix including oligomycin (an ATP synthesis inhibitor), carbonyl cyanide-4 (trifluoromethoxy) phenylhydrazone (FCCP; uncoupling synthesis of ATP from the ETC), and rotenone and antimycin A at the indicated time points (Figure 1G). At rest, NK cells preferentially utilize OXPHOS, and although no difference in the basal OCR levels was observed in the 2 groups (Figure 1H), both maximal respiration rate and spare respiratory capacity (SRC) were significantly reduced in total NK cells from HIV-1^+^ donors (Figure 1, I and J). The total ATP production rates from OXPHOS and glycolysis were consequently lower in the HIV-1 group compared with controls (Figure 1K).

Given that adaptive CD56^dim^CD57^+^NKG2C^+^ NK cells are expanded in HIV-1 infection — as a consequence of more frequent HCMV reactivations and ongoing inflammation, representing a large proportion of the peripheral NK cell pool (Figure 1, A and B) — we sought to determine the relationship between the magnitude of these cells and respiratory capacity. Whereas a positive correlation was observed between the frequencies of adaptive NK cells and maximal respiration in HCMV^+^ controls (Figure 1L) as previously reported (28), this association was not observed in HIV-1^+^ donors, suggesting that the superior metabolic profile of NKG2C^+^ NK cells is diminished in HIV-1 infection (Figure 1L). Adaptive NK cells are less sensitive to innate cytokines but display augmented responses and, in particular, higher IFN-γ production through CD16 activation (12). The relationship between the metabolic fitness and effector response in adaptive NK cells upon CD16 triggering in the cohort as a whole is reflected in the correlation between IFN-γ production and maximal respiration (Figure 1M).

To further investigate the metabolic activity within different NK cell subpopulations, we used a newly developed single-cell metabolic profiling assay (SCENITH), using puromycin for measuring protein synthesis levels (Supplemental Figure 1B) (30). Protein synthesis is a bioenergy-dependent process and tightly correlates with ATP levels, reflecting the global metabolic activity of immune subpopulations. Mitochondrial and glucose dependency were calculated based on the changes in puromycin levels after the addition of OXPHOS inhibitor (oligomycin) and glucose oxidation inhibitor (2-DG), respectively. Consistent with reduced ATP synthesis detected by Seahorse, NK cells from patients with HIV-1 exhibited a trend toward lower levels of energy-intensive protein synthesis (Supplemental Figure 1, C–F). Interestingly, while adaptive NK cells exhibited a trend toward higher mitochondrial and lower glucose dependence compared with canonical NK cells in HIV-1^–^ donors, this was less evident in HIV-1 infection (Supplemental Figure 1G), reinforcing our observed associations (Figure 1L).

### Glycolysis and OXPHOS are required for NK cell IFN-γ production following CD16 crosslinking.

**Figure 2:**
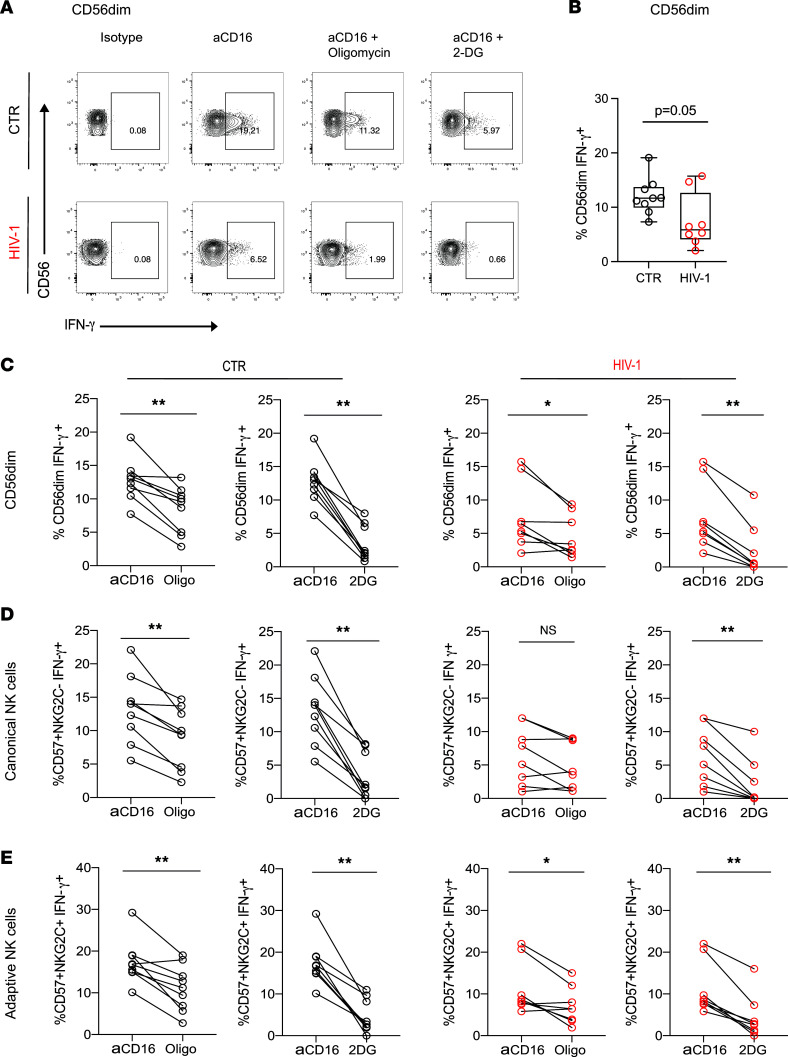
Metabolic requirements of NK cells for IFN-γ production after CD16 activation. (**A**) Representative flow plots illustrating IFN-γ production by CD56^dim^ NK cells from a HCMV^+^HIV-1^–^ control (CTR) and HIV-1^+^ donor when stimulated with plate-bound anti-CD16 antibody alone or in the presence of oligomycin or 2-DG as indicated. (**B**) Summary analysis of IFN-γ production by CD56^dim^ NK cells following anti-CD16 stimulation from *n* = 9 HCMV^+^HIV-1^–^ CTR (black circles) and *n* = 8 patients with chronic HIV-1 (red circles). (**C**–**E**) Paired data showing IFN-γ production from CD56^dim^, canonical, and adaptive NK cells following anti-CD16 stimulation alone or in the presence of oligomycin or 2-DG in CTR donors (black circles) and HIV-1^+^ donors (red circles). Significance determined by 2-tailed Mann-Whitney *U* test or Wilcoxon matched-pairs signed-rank test;*P < 0.05, **P < 0.01.

Upon stimulation, NK cells configure their metabolic machinery to meet their energy demands and synthesize molecules required for NK cell effector function. Depending on the activating stimulus and length of stimulation, NK cells can upregulate glycolysis and OXPHOS (23). In this study, we focused on receptor stimulation via CD16, the engagement of which leads to strong activation of NK cell function and which is thought to represent an important pathway of NK cell antiviral control of HIV-1 infection through recognition of antibody-coated target cells (17, 31). In particular, NK cell ADCC activity has been linked to the protective efficacy of the RV144 vaccine trial and has been implicated in limiting HIV-1 viral replication in various viral control phenotypes (32–34). To investigate the requirement of OXPHOS for receptor-mediated activation of NK cells, we tested the ability of NK cells to produce IFN-γ following anti-CD16 stimulation in the presence or absence of oligomycin. In line with previous observations (4, 35), the mean baseline levels of NK cell IFN-γ production were reduced in HIV-1 infection (Figure 2, A and B). Addition of oligomycin significantly impaired NK cell subset IFN-γ production in response to CD16 triggering in the control group (Figure 2, A and C–E). However, this effect was less prominent in CD56^dim^ and NK cell subsets in HIV-1^+^ individuals, in keeping with an already decreased ability to produce IFN-γ (Figure 2, A and C–E). Of note, the presence of oligomycin did not affect NK cell survival at the concentration used during the 6-hour stimulation (Supplemental Figure 2, A and B). Glycolytic inhibition by addition of the inhibitor 2-DG in the assay further inhibited IFN-γ production by NK cells in both study groups (Figure 2, A and C–E).

Together, these data are consistent with observations that glycolysis and OXPHOS are required for NK cell IFN-γ production following short-term receptor stimulation in humans (36). However, the effect of OXPHOS inhibition was blunted in HIV-1 infection in donors with already-suppressed function at baseline, in whom no correlation was observed between NK cell baseline IFN-γ production and following the addition of oligomycin (Supplemental Figure 2C).

### Adaptive NK cells in HIV-1 infection display evidence of dysfunctional mitochondria.

**Figure 3:**
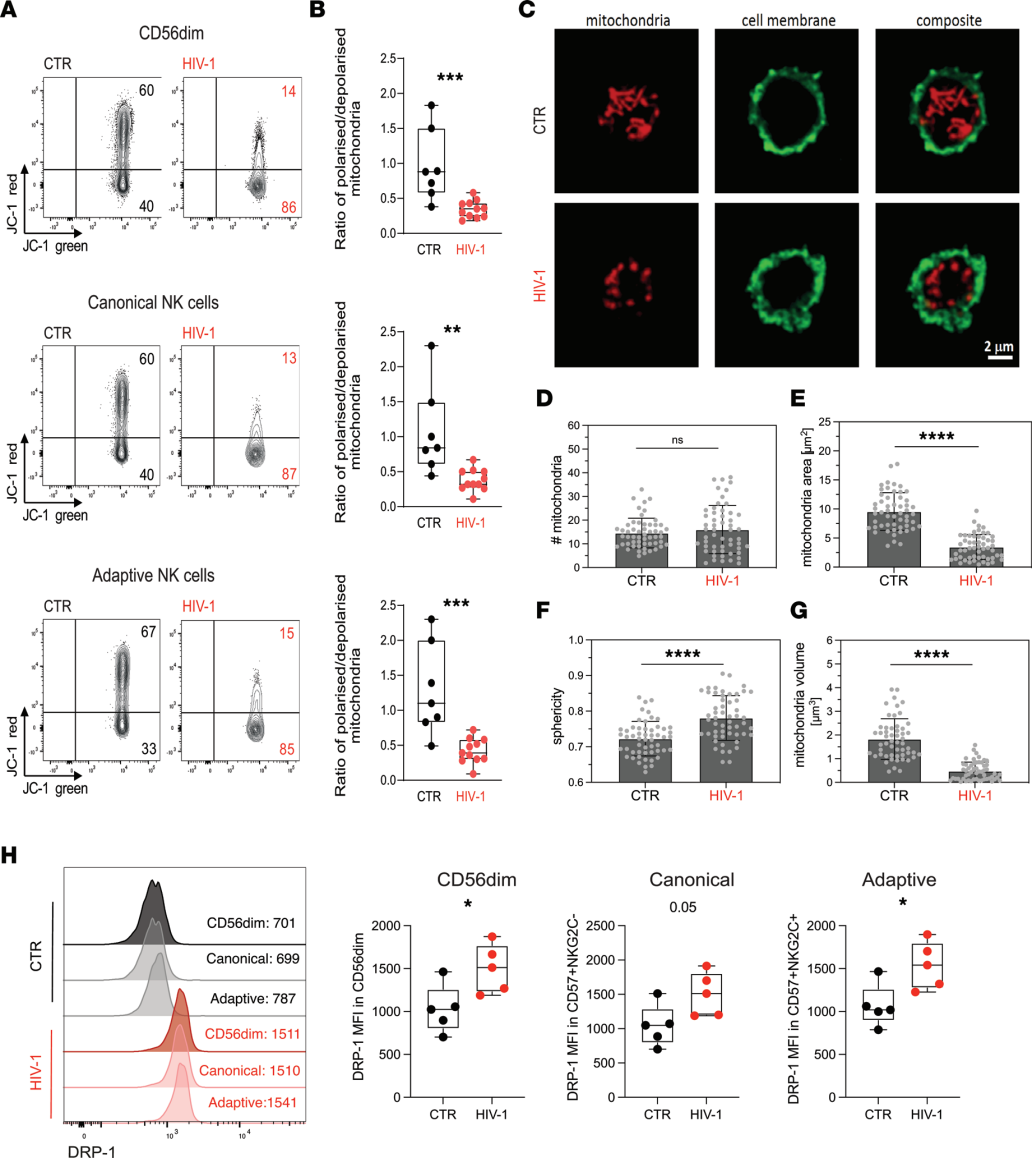
Evaluation of NK cell mitochondrial health. (**A** and **B**) Representative flow plots (**A**) and box-and-whisker plots (**B**) depicting the ratio of polarized over depolarized mitochondrial (mitochondrial membrane potential [ΔΨm]) of total CD56^dim^ NK cells and NK cell subsets by JC-1 staining in HCMV^+^HIV-1^–^ controls (CTR, *n* = 7) and HIV-1^+^ patients (HIV-1, *n* = 11). JC-1 red staining designates polarized mitochondria, while loss of JC-1 red shows depolarization. The box-and-whisker plots show the median, quartiles, and range. (**C**) Representative confocal images of mitochondria (red) from purified NK cells from an HIV^–^ CTR and an HIV-1^+^ donor. The NK cell membrane was visualized by staining with wheat germ agglutinin (green). Scale bar: 2 μm. (**D**–**G**) Quantification of mitochondria numbers, area, sphericity, and volume in purified NK cells from *n* = 3 CTR and *n* = 3 HIV-1^+^ donors. Each symbol represents the mean value of 1 cell. Data are from a minimum of 50 cells from 3 independent donors. (**H**) Representative histograms and summary data of DRP-1 expression levels in CD56^dim^, canonical (NKG2C^–^CD57^+^), and adaptive (NKG2C^+^CD57^+^) NK cells (CTR, *n* = 5; HIV-1, *n* = 5). Significance was determined by 2-tailed Mann-Whitney *U* test or 1-way ANOVA with Tukey’s post-hoc test; *P < 0.05, **P < 0.01, ***P < 0.001, ****P < 0.0001.

The decrease in SRC in NK cells from HIV-1^+^ donors suggest a reduced ability of these cells to produce energy in response to stress or increased work demands and prompted us to further investigate the mitochondrial health of NK cells. In particular, greater SRC is shown to correspond to enhanced mitochondrial fitness, a feature of adaptive NK cells and memory CD8 T cells (28, 37). We, therefore, utilized the ratiometric fluorescent dye JC-1 to assess further the mitochondrial membrane potential (ΔΨm) of NK cells, which represents a key readout of mitochondrial function. NK cells from HIV-1^+^ donors displayed a lower ΔΨm, especially within adaptive subsets, compared with NK cells from HIV-1^–^ donors (Figure 3, A and B). This is exemplified by the lower red/green fluorescence intensity ratio of JC-1, which does not depend on mitochondrial shape, size, or density, suggesting that mitochondrial dysfunction in NK cells — especially of adaptive subpopulations that are enriched in HIV-1^+^ donors — could influence their cellular fitness and effector function. Evaluation of the ΔΨm by analyzing tetramethylrhodamine, methyl ester (TMRM) staining was consistent with JC-1 (Supplemental Figure 3, A and B).

Mitochondria are dynamic organelles that configure their morphology and number to regulate their function and distribution (38). To further interrogate the relationship between mitochondrial dysfunction and mitochondrial morphological changes, we performed confocal imaging. Isolated NK cells from 3 HIV-1^–^ controls and 3 HIV-1^+^ donors with evidence of depolarized mitochondria were stained with MitoTracker Deep Red, which accumulates in active mitochondria and enables localization. Our results demonstrate that, despite no difference in the number of mitochondria (Figure 3, C and D), NK cells from HIV-1^+^ individuals exhibited mitochondrial structural differences, adopting a change in their configuration from a long tubular shape into shrunken small spherical shapes (Figure 3, C and E–G, and Supplemental Videos 1 and 2). These data suggest that loss of ΔΨm in NK cells in HIV-1 infection most likely triggers a mitochondrial transformation related to dysfunction (39). Mitochondrial fragmentation is driven by the cytosolic GTPase dynamin-related protein 1 (DRP-1), which induces mitochondrial fission by translocating to the outer mitochondrial membrane, while optic atrophy 1 (OPA-1) supports mitochondrial fusion (40). Interestingly, although OPA-1 expression remained similar between the 2 study groups (Supplemental Figure 4A), NK cells from HIV-1^+^ donors displayed higher levels of DRP-1 protein, consistent with the increase in mitochondrial fission (Figure 3H). Structural changes in the mitochondria have been related to bioenergetic insufficiency and increased mitochondrial sphericity in T cells in the absence of costimulation (41), and they could also be a reflection of oxidative damage in the cell (42). However, analysis of reactive oxygen species (ROS) levels in NK cells from patients with chronic HIV-1 infection compared with those of NK cells from HCMV^+^HIV-1^–^ controls did not show a significant increase in the levels of ROS in HIV-1 infection (Supplemental Figure 4B). This could be due to a rescue mechanism, with adaptive NK cells previously described to display elevated levels of BCL-2, which may be important in limiting oxidative stress (28, 43).

The enhanced oxidative metabolism observed in adaptive NK cells from HCMV^+^HIV-1^–^ donors has been reported to depend partly on increased expression of *ARID5B* and induction of genes encoding components of the ETC, including *UQCRB*, the ETC complex III gene (28). To determine whether there were any differences in the expression of *ARID5B* or *UQCRB* in isolated total NK cells from HCMV^+^ controls and HIV-1^+^ individuals, we performed quantitative PCR (qPCR). We found increased levels of *ARID5B* expression in NK cells from HIV-1^+^ donors relative to controls (Supplemental Figure 4C). We did not detect any differences in the levels of *UQCRB* in a small number of donors tested (Supplemental Figure 4D). The ex vivo frequency of adaptive NK cells correlated positively with *ARID5B* levels of expression, showing greater expansions of adaptive NK cells in HIV-1 infection, relative to controls (Supplemental Figure 4E). Our findings, therefore, suggest that mitochondrial structural disorganization rather than a decreased expression of *ARID5B* could affect the performance of the ETC and metabolic adaptation of NK cells in HIV-1 infection.

### Treatment with IL-15 compensates for the metabolic defects of NK cells in HIV-1 infection.

**Figure 4:**
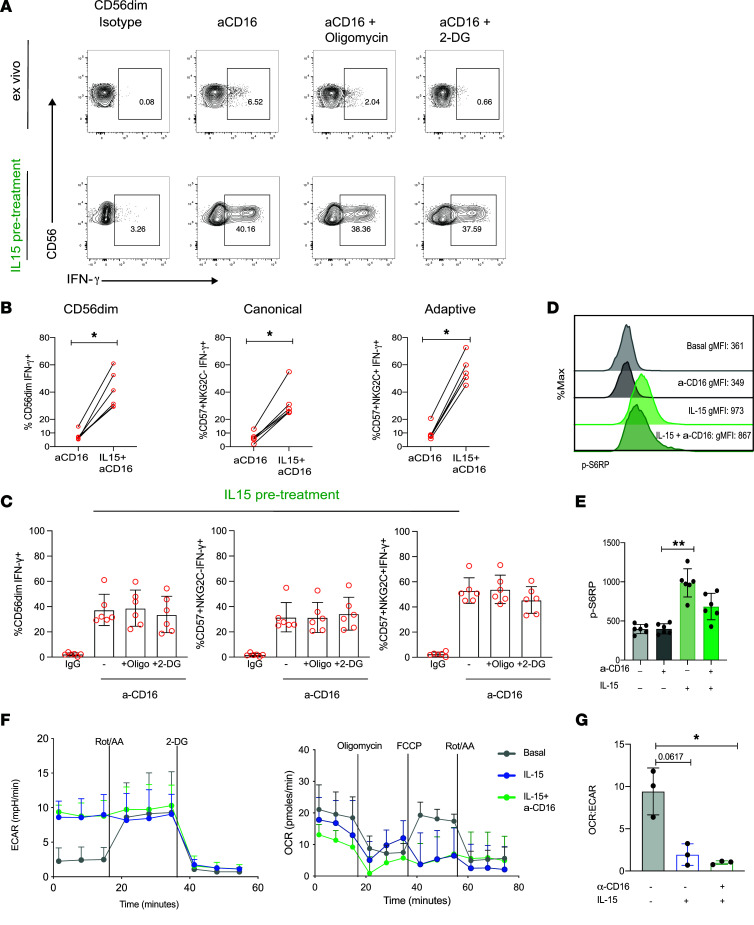
IL-15 treatment ameliorates NK cell metabolic requirements for receptor stimulation in HIV-1 infection. (**A**) Representative flow plots showing IFN-γ production by CD56^dim^ NK cells from an HIV-1^+^ donor following stimulation with anti-CD16 with or without IL-15 pretreatment and in the presence or absence of the following inhibitors: oligomycin or 2-DG. The representative ex vivo flow panel corresponds to the same HIV-1 donor depicted in Figure 2A (bottom panel), showing paired responses to ex vivo stimulation with anti-CD16 and following IL-15 pretreatment within the same donor. (**B**) Paired analysis showing IFN-γ production ex vivo and following IL-15 priming in response to anti-CD16 stimulation from NK cells (*n* = 6). Ex vivo: 6-hour stimulation with anti-CD16; IL-15 pretreatment: incubation with IL-15 for 48–72 hours followed by 6-hour anti-CD16 stimulation. (**C**) Summary data showing IFN-γ production in response to isotype control or anti-CD16 stimulation, following IL-15 pretreatment, in the presence of oligomycin or 2-DG by CD56^dim^, canonical, and adaptive NK cells from *n* = 6 HIV-1^+^ donors. Data are shown as mean ± SD. (**D** and **E**) Representative histograms and summary bar charts of pS6 expression levels to evaluate mTORC1 activity after stimulation in CD56^dim^ NK cells from *n* = 6 HIV-1^+^ individuals. (**F**) Real-time analysis of aerobic glycolysis (ECAR) and basal oxygen consumption rate (OCR) in purified NK cells from *n* = 3 HIV-1^+^ donors in the presence or absence of anti-CD16 and/or IL-15 pretreatment. (**G**) OCR/ECAR ratio in NK cells from HIV-1^+^ individuals. Significance was determined by Wilcoxon matched-pairs signed-rank test and paired *t* test for **G**. One-way ANOVA with multiple-comparison test was performed for **C** and **E**; *P < 0.05, **P < 0.01.

IL-15 plays a vital role in NK cell survival and differentiation and in priming NK cells for enhanced effector function in vivo (44). We, therefore, investigated whether IL-15 priming in vitro could improve NK cell responses to stimulation via CD16 by circumventing the metabolic requirements for efficient receptor-mediated activation in HIV-1^+^ donors. Pretreatment with IL-15 for 48–72 hours, followed by anti-CD16 stimulation, led to a striking increase in IFN-γ production by total CD56^dim^ NK cells but also canonical and adaptive subsets (Figure 4, A and B). Inhibition of OXPHOS with oligomycin in primed NK cells did not affect IFN-γ production in response to receptor stimulation, while a small nonsignificant difference was observed in the presence of 2-DG (Figure 4C).

IL-15 is known to stimulate multifaceted metabolic activities during the maturation of NK cells via the mTORC1, a key regulator of cellular metabolism (20). IL-15 signaling and activation of NK cells leads to enhanced glucose uptake and enhanced functionality. Consistent with this, IL-15 priming of NK cells from HIV-1^+^ donors prior to anti-CD16 stimulation induced greater phosphorylation of the S6 ribosomal protein (pS6), consistent with enhanced mTORC1 signaling in CD56^dim^ NK cells (Figure 4, D and E).

Next, to explore the effect of IL-15 priming on mitochondrial health, we examined the mitochondrial polarization by analyzing TMRM staining in a subset of people with HIV-1 (*n* = 3) (Supplemental Figure 5A). Oligomycin, which induces maximal mitochondrial polarization, was used as a positive control, while FCCP, which induces mitochondrial membrane depolarization, was used as a negative control (45). Compared with untreated cells, IL-15 priming induced a higher ΔΨm in NK cell subsets in HIV-1 infection (Supplemental Figure 5A). To further evaluate the metabolic effects of IL-15 pretreatment in the presence or absence of anti-CD16 stimulation in isolated NK cells from HIV-1^+^ patients, we performed extracellular flux assays to directly analyze metabolic responses. Not surprisingly, NK cells treated with IL-15 displayed a higher basal ECAR relative to resting NK cells, without, however, a significant increase in basal OCR (Figure 4F). A decreased dependence on OXPHOS was observed in IL-15–treated NK cells, especially in the presence of CD16 triggering, as evidenced by the decreased ratio of OCR/ECAR compared with basal levels (Figure 4G). The shift to glycolysis was confirmed by the SCENITH assay in all NK cell subsets, demonstrating increased glycolytic capacity and decreased mitochondrial dependence (Supplemental Figure 5, B and C), in keeping with the Seahorse observations. Overall, these data suggest that enhanced NK cell function does not hinge on improved mitochondrial health and are in line with findings showing that IL-15 priming can increase mitochondrial fragmentation in NK cells (46).

The observed higher levels of expression of DRP-1 in NK cells in HIV-1 infection, which promotes mitochondrial fission, prompted us to investigate further the effect of small molecule inhibitors of mitochondrial fission, Mdivi-1 and fusion promoter, M1, in improving IFN-γ production to anti-CD16 stimulation. Pharmacological manipulation of mitochondrial dynamics led to a small increase in effector function and partial restoration of IFN-γ production when used in conjunction with anti-CD16 stimulation (Supplemental Figure 5D).

**Figure 5:**
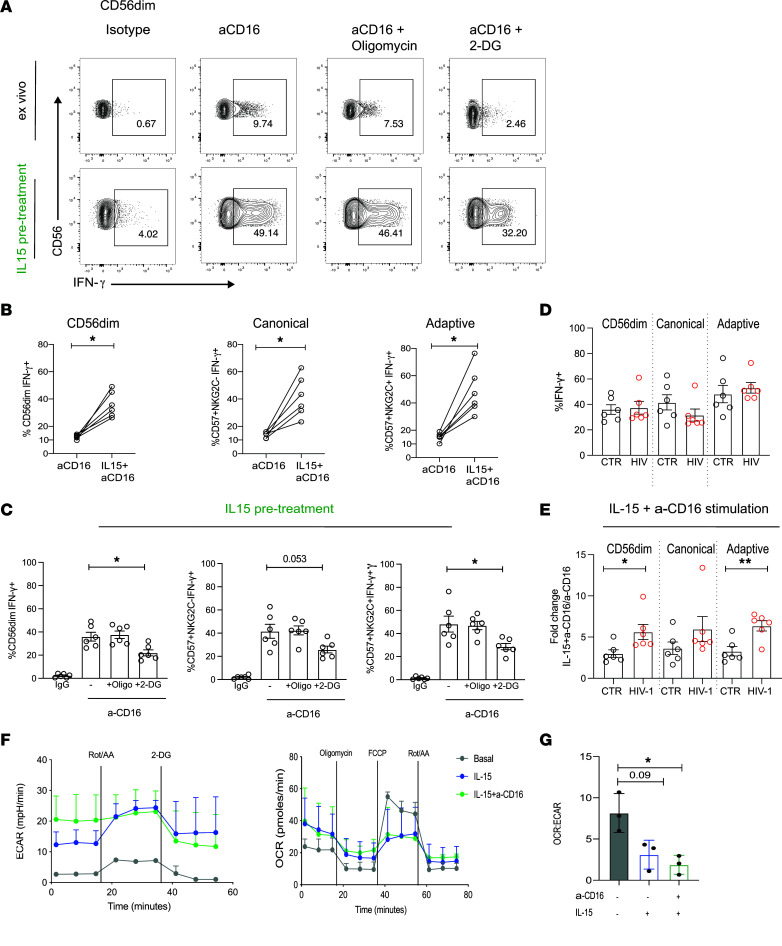
Effect of IL-15 priming on NK cells from HCMV^+^HIV-1^–^ donors. (**A**) Representative flow plots showing IFN-γ production by CD56^dim^ NK cells from a HCMV^+^HIV-1^–^ donor following stimulation with anti-CD16 with or without IL-15 pretreatment and in the presence or absence of oligomycin or 2-DG. (**B**) Paired analysis showing IFN-γ production ex vivo and following IL-15 priming in response to anti-CD16 stimulation from total CD56^dim^, canonical, and adaptive NK cells (*n* = 6). Ex vivo: 6-hour stimulation with anti-CD16; IL-15 pretreatment: incubation with IL-15 for 48–72 hours followed by 6-hour anti-CD16 stimulation. (**C**) Summary data showing IFN-γ production in response to isotype control or anti-CD16 stimulation, following IL-15 pretreatment, in the presence of oligomycin or 2-DG by CD56^dim^, canonical, and adaptive NK cells from *n* = 6 control donors. (**D**) Summary bar charts showing IFN-γ production in CD56^dim^, canonical, and adaptive NK cells following IL-15 priming, in both HIV-1^+^ and control donors, in response to anti-CD16 stimulation. (**E**) Fold change in IFN-γ production to anti-CD16 stimulation between IL-15 pretreated and directly ex vivo stimulated NK cells. (**F**) Real-time analysis of aerobic glycolysis (ECAR) and basal oxygen consumption rate (OCR) in purified NK cells from *n* = 3 HIV-1^–^ donors in the presence or absence of anti-CD16 and/or IL-15 pretreatment. (**G**) OCR/ECAR ratio in NK cells from HIV-1^–^ individuals. Significance determined by 2-tailed Mann–Whitney *U* test (**D** and **E**) , Wilcoxon matched-pairs test (**B**), or paired *t* test for **G**. One-way ANOVA with multiple-comparison test was performed for **C**; *P < 0.05, **P < 0.01.

Next, we assessed the effect of IL-15 priming on NK cell IFN-γ production in response to anti-CD16 stimulation in HIV-1^–^ donors. Similarly to HIV-1^+^ donors, pretreatment with IL-15 significantly increased responses in all NK cell subsets (Figure 5, A and B). Although oligomycin had no effect on IFN-γ production, a small reduction in IFN-γ was observed following the addition of 2-DG (Figure 5C). IL-15 priming produced similar levels of IFN-γ by NK cells following receptor stimulation, irrespective of HIV-1 status (Figure 5D), showing functional restoration of low-level ex vivo IFN-γ responses in HIV-1 infection to levels equivalent to that observed for HIV-1^–^ donors following IL-15 pretreatment. Interestingly, the fold change increase in IFN-γ production by NK cells, especially adaptive subsets, was higher in HIV-1 infection following IL-15 pretreatment (Figure 5E). As expected, stimulation with IL-15 upregulated glycolysis (ECAR), and similarly to observations in HIV-1 infection, there was a decreased OCR/ECAR ratio and reliance on oxidative metabolism (Figure 5, F and G).

## Discussion

Burgeoning evidence indicates that metabolic plasticity is important for fine-tuning immune cell function. Whereas most studies have focused on T cell metabolism during human chronic viral infections, much less is known about the metabolic lifestyle of NK cells in this setting. In this study, we assessed the metabolic profile of NK cell subsets and requirements for production of IFN-γ following receptor stimulation, via CD16 crosslinking, during chronic HIV-1 infection. Our results implicate dysregulated metabolism as an upstream driver of NK cell functional fate. NK cells in viremic HIV-1 infection are characterized by impaired OXPHOS, reduced metabolic reserve, and mitochondrial defects. IL-15 pretreatment effectively eliminated the metabolic requisite for responses to receptor stimulation, boosting NK cell responses to similar levels as those observed in the HIV-1^–^ group.

In general, resting NK cells are quiescent, as evidenced by the low basal levels of glycolysis and OXPHOS. However, NK cells in chronic HIV-1 infection display an inability to rely on mitochondrial function under conditions of increased energy demands, as evidenced by the reduction in both maximal respiration and SRC. Although we did not formally test the metabolic profile of adaptive CD57^+^NKG2C^+^ NK cells in this study via Seahorse, due to sample limitations and the potential effect of sorting on the redox state of the cells (47), these cells are enriched and represent a sizeable proportion of the peripheral NK cell pool in HIV-1 infection. Notably, there was no correlation between CD57^+^NKG2C^+^ NK cells from HIV-1^+^ donors and maximal respiration, and these populations exhibited lower mitochondrial dependence compared with HIV-1^–^ donors. This suggests that NK cells in HIV-1 infection do not acquire the characteristic metabolic profile defining HCMV adaptive (CD57^+^NKG2C^+^) NK cells in HIV-1^–^ individuals with NK cell memory-like increased OXPHOS (28). Alternatively, adaptive subpopulations expressing NKG2C could gradually lose their bioenergetic advantage with progressive HIV-1 infection as a consequence of persistent activation and evolving dysregulation. We did not detect significant differences in the rates of glycolysis or glycolytic reserve between the 2 study groups. However, further validated glucose transport assays are required to confirm these results prior to excluding any formal defects in glucose uptake (48).

Upon stimulation, NK cells configure their metabolic machinery to meet their energy demands and synthesize molecules required for NK cell effector function. Depending on the activating stimulus and length of stimulation, NK cells can upregulate glycolysis and OXPHOS (24). In this study, we focused on receptor stimulation via CD16, a potent activator of NK cell function and an important means of NK cell antiviral control in HIV-1 infection. NK cells required OXPHOS and glycolysis to produce IFN-γ in the control group. Similar effects were observed in murine models utilizing NK1.1 and Ly49D activating receptor stimulation (25) and in humans following CD16 stimulation (36), supporting the notion that OXPHOS is a metabolically required second signal for IFN-γ production mediated via ITAM-coupled adapters. However, the effect of OXPHOS inhibition was less pronounced in individuals living with HIV-1 who exhibited suppressed OXPHOS and responses to receptor stimulation via CD16. Additional work is required to fully elucidate the metabolic plasticity of NK cells in HIV-1 infection, including the role of alternative fuels and how perturbations in nutrient availability during HIV-1 infection may affect NK cell functionality.

Metabolic inflexibility has been described to underline dysfunctional HIV-specific CD8 T cells (15). In particular, the polyfunctionality of CD8 T cells from spontaneous HIV-1 controllers was more reliant on mitochondrial function rather than glycolysis, and differences in glucose dependency described in SIV-specific CD8 T cells have been found to correlate with their capacity to suppress SIV infection. More recently, we described that terminally exhausted virus-specific CD8 T cells in HIV-1 infection are characterized by impaired OXPHOS and mitochondrial defects (16). Interestingly, the role of mitochondrial fitness was recently highlighted as a critical component of NK cell function and antiviral responses during acute retrovirus infection (45, 46). We, therefore, reasoned that the observed inability of NK cells to utilize OXPHOS during HIV-1 infection could reflect dynamic changes in the mitochondria, which are essential hubs of metabolic activity determining the function and fate of immune cells. A global mitochondrial defect was supported by decreased ΔΨm in NK cells in HIV-1 infection. Notably, isolated NK cells from HIV-1^+^ patients displayed marked changes in mitochondrial architecture characterized by the presence of small spherical mitochondria and increased levels of DRP-1, the mitochondrial fission regulator (40). The shape of the mitochondria may directly affect their bioenergetic function, with elongated mitochondria being associated with more efficient ATP production and with mitochondrial dysfunction resulting in fragmentation described in a number of pathological conditions (49). The delicate balance between mitochondrial fusion and fission controls mitochondrial shape, number, and size and is important for function (respiratory capacity) and quality control of mitochondria, raising the possibility that alterations in mitochondrial dynamics in HIV-1 infection could influence NK cell metabolic reprogramming. Along these lines, pharmacological manipulation of mitochondrial dynamics in combination with anti-CD16 stimulation led to partial restoration of NK cell function in HIV-1 infection. This is also in keeping with observations that mitochondrial fragmentation, induced by sustained activation of the Drp1 signaling pathway, impaired metabolic efficiency and effector function of tumor-infiltrating NK cells, whereas inhibiting mitochondrial fission enhanced oxidative metabolism and NK cell–mediated tumor surveillance (50). Similarly, pharmacological enforcement of mitochondrial fusion promoted oxidative capacity and superior functionality in effector T cells (51). It would be important to dissect further in future studies the intricate relationship between the modulation of mitochondrial shape and NK cell energetic state and the downstream cellular processes, such as mitophagy (52). Such investigations will provide a framework to study these crucial biological interactions on the metabolic regulation of NK cell subsets during chronic viral infections.

The observed alterations in the metabolic machinery of NK cells during chronic HIV-1 infection influencing their mitochondrial integrity bears similarities to exhausted CD8 T cells. In particular, exhausted CD8 T cells during chronic viral infections show extensive mitochondrial alterations with an impaired ability to use mitochondrial energy supply coupled to the programmed death-1 (PD-1) pathway and exhibiting an EOMES^hi^Tbet^lo^TIGIT^+^ phenotype (16, 53–55). Signaling via PD-1 promoting exhaustion of CD8 T cells has been found not only to affect mitochondrial morphology but also to repress the key metabolic transcriptional regulator peroxisome proliferator-activated receptor-γ (PPAR-γ) coactivator 1a (PGC-1a), controlling energy metabolism and mitochondrial biogenesis and suppressing OXPHOS and glycolysis in virus-specific T cells (55). Upregulation of inhibitory molecules on NK cells could also serve as a pathway suppressing their metabolic fitness. Recently, it was shown that chronic stimulation of adaptive NKG2C^+^ NK cells in vitro led to a marked induction of checkpoint inhibitory receptors PD-1 and lymphocyte activation gene-3 (LAG-3) and led to sharing of epigenetically driven programs with exhausted CD8 T cells (14). Moreover, a role for LAG-3 in regulating CD4 T cell metabolism and mitochondrial biogenesis has been identified (56). We have previously reported a modest increase in the levels of PD-1 expression on NK cells in HIV-1 infection (4); however, the role of inhibitory receptors in controlling NK cell metabolism remains to be further defined.

Pretreatment of NK cells with IL-15 alleviated the need for OXPHOS for CD16 receptor–stimulated IFN-γ production, extending observations from murine models (25). Our results imply that IL-15 increased mTORC1 activity, an important regulator of NK cell metabolism, contributing to the upregulation of glycolysis (20). Metabolic regulation of IFN-γ production could reflect a switch to a posttranscriptional regulatory mechanism for IFN-γ production as described for T cells, where GAPDH has been described to act as a translation repressor, binding to *Ifng* 3′UTR (57). However, a recent report showed that engagement of the activating receptor NK1.1 in high-dose IL-15–primed murine NK cells imparted a unique transcriptional profile, identifying c-Myc and its targets to be positively associated with *Ifng* transcription. In this setting, cytokine-primed NK cells no longer required a metabolism-derived additional signal for IFN-γ production (58). Notably, IL-15 priming increased chromatin accessibility with enrichment for AP-1 motifs associated with enhanced function in HCMV adaptive NK cells (59). It is plausible that the broader epigenetic reconfiguration induced by IL-15 could alter downstream ITAM signaling recovering NK cell function in HIV-1 infection, but the underlying mechanism requires further investigation.

The effects of IL-15 priming on NK cell metabolism and functionality have important implications for clinical translation in HIV-1 infection. The beneficial effects of IL-15 immunotherapy and IL-15 superagonist N-803, capable of activating both NK cells and CD8 T cells and promoting migration to B cell follicles, have been highlighted in vivo in nonhuman primate models (60, 61). Such an approach has been tested in a phase I clinical trial to facilitate the clearance of latent HIV-1 reservoirs (NCT02191098), demonstrating safety and tolerability (61). The ability of IL-15 to enhance ADCC and augment NK cell–mediated killing of HIV-infected target cells ex vivo (62) may prove vital in the development of a functional cure for HIV-1. Our findings, together with recent reports of IL-15–mediated metabolic reprogramming alone or in combination with mitochondrial-targeted treatment improving the efficacy of HIV-specific CD8 T cells from noncontrollers (15, 16) highlight the complementary effects of such an approach to simultaneously reinvigorate multiple arms of the immune response. However, such approaches must be carefully designed, considering the potential detrimental effect of continuous IL-15 treatment driving metabolic exhaustion of NK cells (63).

In summary, our results demonstrate that NK cells in HIV-1 infection exhibit metabolic defects affecting both canonical and adaptive subsets underlined by mitochondrial dysfunction/structural alterations and bearing similarities to exhausted CD8 T cells described in HIV-1 and other chronic viral infections. These findings identify mitochondrial-centered dysfunction as a key area for future research and as a promising target for future combined reconstitution therapies. IL-15 pretreatment bypasses the metabolic requirement for OXPHOS upon CD16-mediated activation and potentiates NK cell function, which is of considerable clinical interest in the field of HIV-1 as a powerful multipronged therapeutic strategy. Further insights into the potential role of inhibitory receptors will provide us with a more comprehensive understanding of the integration of signals and the role of metabolism in fine-tuning the range and potency of NK cell antiviral functions.

## Methods

### Patients.

Cryopreserved peripheral blood mononuclear cells (PBMCs) from 11 HIV-1, HCMV-seropositive treatment naive patients sampled during chronic infection were utilized (mean age = 40.5, range = 27–49; mean log viral load (logVL) = 4.89, range = 4.14–6.33; mean CD4 count 475 cells/mL, range = 61–720). Participants were recruited at the Mortimer Market Centre for Sexual Health and HIV Research and the Royal Free Hospital (London, United Kingdom) following written informed consent as part of a study approved by the Berkshire Research Ethics Committee (REC 16/SC/0265). Eleven demographically age- and sex-matched HCMV-seropositive HIV-1^+^ donors (mean age = 37.1, range = 26–49) with sizeable adaptive NK cell responses (>10%) were used for comparison (Figure 1, A and B), from whom blood was taken and cryopreserved for later use with written informed consent in accordance with the Declaration of Helsinki. All study participants were negative for anti-Hepatitis C virus antibody and anti-HBsAg, and they were HCMV seropositive. HCMV serology was determined at University College London Hospital clinical virology lab by the ARCHITECT CMV IgG assay (AU/mL) (Abbott Diagnostics).

### Flow cytometric analysis.

The following fluorochrome-conjugated antibodies were used in this study: CD16 BB700 (3G8, BD Biosciences, catalog 746199, dilution 1 in 200), CD56 BV605 (NCAM16.2, BD Biosciences, catalog 562780, dilution 1 in 50), CD57 BV421 (NK-1, BD Biosciences, catalog 563896, dilution 1 in 200), CD3 BV650 (OKT3, BioLegend, catalog 317324, dilution 1 in 100), CD14 BV510 (M5E2, BioLegend, catalog 301842, dilution 1 in 200), CD19 BV510 (HIB19, BioLegend, catalog 302242, dilution 1 in 200), CD56 PE Dazzle (HCD56, BioLegend, catalog 359620, dilution 1 in 100), CD16 PerCP (3G8, BioLegend, catalog 302029, dilution 1 in 50), NKG2C PE or Alexa Fluor 700 (134591, R&D systems, catalog FAB138P-100, dilution 3 in 50) for surface antigens and IFN-γ PE-CY7 (B27, BD Biosciences, catalog 557643, dilution 1 in 100) and pS6 AlexaFluor647 (D57.2.2E, Cell Signaling Technology, catalog 14733S, dilution 1 in 100) for intracellular antigens. Briefly, cryopreserved PBMC were washed in PBS and surface stained at 4°C for 20 minutes with saturating concentrations of different combinations of antibodies in the presence of fixable live/dead stain (Invitrogen). Cells were then fixed and permeabilized for the detection of intracellular antigens. To assess OPA-1 and DRP-1 expression, DRP-1 FITC (Polyclonal, Bio-Techne, catalog NB110-55288F, dilution 1 in 100), OPA1 (D7C1A, Cell Signaling Technology, catalog 67589S, dilution 1 in 100), and Alexa Fluor 488 secondary antibodies (Abcam, catalog ab150077, dilution 1 in 1000) were used. Assessment of mitochondrial membrane polarization and ROS production was performed by incubation with 2 μM JC-1, 50 nM TMRE (Thermo Fisher Scientific), or 5 μM MitoSOX (Invitrogen), according to the manufacturer’s instructions. Samples were acquired on a BD Fortessa X20 using BD FACSDiva8.0 (BD Bioscience), and data were analyzed using FlowJo 10 (Tree Star Inc.). Supplemental Figure 1A and Figure 1A include the gating strategy for the identification of NK cell subsets into adaptive and canonical on the basis of expression of CD57 and NKG2C.

### NK cell isolation.

NK cells were enriched from PBMCs using a negative-selection magnetic bead kit (Miltenyi Biotec) as per the manufacturer’s instructions (>94% purity and viability).

### Extracellular flux assays.

Extracellular flux assays were performed using XFp Analyzer (Agilent Technologies). Purified NK cells were seeded at 2 × 10^5^ cells per well in Seahorse cell plates coated with CellTak (Corning). Mitochondrial OXPHOS was evaluated using the mitostress test kit and glycolysis using the glycolytic rate assay kit (Agilent Technologies). The OCR and ECAR were measured in XF RPMI medium supplemented with 10 mM glucose, 1 mM pyruvate, and 2 mM glutamine in response to oligomycin (1 μM), FCCP (1 μM), rotenone/antimycin A (0.5 μM), and 2-DG (50 mM) (Agilent Technologies). To evaluate the effect on the metabolic profile of IL-15 pretreatment, NK cells were purified and cultured with 100 ng/mL of IL-15 for 48 hours before stimulation with CD16-coated beads (Miltenyi Biotec) or left in IL-15 supplemented media. Maximum respiration is the average OCR valueafter FCCP injection. The SRC was calculated as the average post-FCCP injection minus basal OCR average. Glycolytic reserve was calculated as average postrotenone/antimycin A injection ECAR values minus basal ECAR values. Total ATP production rate was calculated as the sum of glycolytic and oxidative ATP production (64). Briefly, for ECAR, the total change in media pH was converted into proton efflux rate (PER_total_) and then subdivided into 2 components: PER from glycolytic lactate production (PER_glyco_) and PER from mitochondrial respiration (PER_mito_). For OCR, mitochondrial OCR (OCR_mtio_) was defined as total OCR (OCR_tot_) minus the OCR in the presence of the respiratory chain poisons rotenone and antimycin (OCR_ROT/AA_), and the value was used to calculate PER_mito_ after consideration of the CO_2_ contribution factor (CCF). Glycolytic ATP production rate is equivalent to Glycolytic Proton Efflux Rate. Oxidative ATP production was calculated as the total OCR minus the oligomycin-OCR (OCR_oligo_). Extracellular flux assays were performed in HIV-1^+^ patients with sufficient available PBMC to enable NK cell isolation.

### SCENITH assay.

The SCENITH assay (SCENITH reagents kit; www.scenith.com) was performed as previously described (30). Briefly, 0.5 × 10^6^ to 1 × 10^6^ cells plated in a 96-well plate, were treated with control, 2-DG (100 mM), oligomycin (Oligo; 1 μM), or a combination of the inhibitors at the same final concentrations, for 30–45 minutes at 37°C. Harringtonine (Neg; 2 μg/mL) was used as a negative control. Following treatment with metabolic inhibitors, puromycin (Puro; 10 μg/mL) was added during the last 30 minutes. Cells were then washed with cold PBS and stained with cell viability dye (Invitrogen) for 15 minutes. Following incubation, cells were washed and stained for extracellular markers before fixation and permeabilization using eBioscience Foxp3 fixation and permeabilization buffer kit (Thermo Fisher Scientific). Intracellular staining of puromycin was then performed for 1 hour in diluted (10×) permeabilization buffer at 4°C.

Calculations used for SCENITH parameters include the following:

(a) Glucose dependence = 100 (Control – 2DG)/(Control – [2-DG + Oligo]); (b) mitochondrial dependence = 100 (Control – Oligo)/(Control – [2-DG + Oligo]); and (c) glycolytic capacity = 100 − mitochondrial dependence.

### Functional assays.

For activation via CD16 crosslinking, 96-well flat-bottom plates (Nunc) were coated with 5 μg/mL anti–human CD16 (clone 3G8, catalog 556617, BD Biosciences) or an isotype-matched control antibody (mIgG1κ, catalog 556648, BD Biosciences) overnight at 4°C. Plates were washed with sterile PBS before addition of 4 × 10^5^ PBMC per well. To assess fuel flexibility, cells were incubated for 6 hours in the presence or absence of 100 nM oligomycin and 50 mM 2-DG (MilliporeSigma). GolgiStop (containing monensin, 1/1,500 concentration) (BD Biosciences) and GolgiPlug (containing brefeldin A, 1/1,000 final concentration) (BD Biosciences) were added for the last 5 hours of culture. Following incubation, cells were washed and stained for extracellular receptors before permeabilization and intracellular staining for IFN-γ (B27, BD Biosciences, catalog 557643, dilution 1 in 100) and pS6 (D57.2.2E, Cell signaling, catalog 14733S, dilution 1 in 100). Where indicated, cells were pretreated with 100 ng/mL IL-15 (Miltenyi Biotec) for 48–72 hours followed by 6-hour stimulation with anti-CD16. In Figures 4 and 5, a paired analysis was performed comparing NK cell responses with anti-CD16 stimulation (ex vivo) and after IL-15 treatment. Some of the ex vivo data presented in Figure 2 were included in Figure 4 (representative matched data).

### Confocal microscopy.

Confocal imaging was performed with an Olympus FV1200 inverted microscope (Olympus) equipped with a 60× 1.4 NA oil-immersion objective at room temperature.

Prior to confocal imaging, purified NK cells were incubated with 50 nM MitoTracker Deep Red FM (Thermo Fisher Scientific) for 60 minutes at 37°C to visualize the mitochondria. After incubation, the cells were plated onto a 0.01 % Poly-L-Lysine–coated 8-well IBIDI chamber for 15 minutes, followed by fixation with 4% PFA/PBS for an additional 30 minutes at room temperature. Additional labeling with 10 μg/mL WGA-Alexa Fluor 488 (Thermo Fisher Scientific) for 10 minutes to visualize the cell membrane was performed. Images were taken with a 250 nm *Z* stepping. Postprocessing and segmentation (TrakEM2 plugin) of fluorescence images was done with ImageJ (NIH). The numbers of mitochondria, surface area, mitochondria volume, and sphericity were obtained by 3D surface rendering of confocal images in Imaris version 9.3 (Bitplane).

### Quantification of gene expression.

Total NK cells were lysed following isolation using MACS beads (Miltenyi Biotec). Total RNA was extracted using RNeasy micro kit (Qiagen) following the manufacturer’s instructions. cDNA synthesis was performed using the high-capacity cDNA reverse transcriptase kit (Applied Biosystems). Real-time PCR was performed using 5 ng of CDNA, fast SYBR green master mix (Applied Biosystems), and the following primers: GAPDH forward, 5′-CCTGCACCACCAACTGCTTA-3′, and reverse, 5′-GGCCATCCACAGTCTTCTGAG-3′; RPLP0 forward, 5′-GCAATGTTGCCAGTGTCTG-3′, and reverse 5′-GCCTTGACCTTTTCAGCAA-3′; and ARIDB5 forward, 5′-CTGAGCCTCTCCCAGCAGCA-3′, and reverse, 5′-CGCCTCCTCTGCCACCTTCT-3′ on a 7500 fast real-time PCR system (Applied Biosystem). GAPDH and RPLP0 were used as housekeeping genes for normalization. Data were analyzed using the ΔCt method relative to housekeeping genes for both control and HIV-1^+^ patients and were expressed as log_2_ expression value.

### Statistics.

Prism 8 (GraphPad Software) and OriginPro 9.1 (OriginLab) were used for statistical analysis as follows: the 2-tailed Mann-Whitney *U* test or 2-tailed Student’s *t* test were used for single comparisons of 2 independent groups, the Wilcoxon signed-rank test or Student’s paired *t* test were used to compare 2 paired groups. For 3 or more groups, Kruskal-Wallis 1-way ANOVA with Dunn’s multiple-comparison test was performed with a nonparametric distribution. The nonparametric Spearman test was used for correlation analysis. To analyze the confocal imaging results, samples were tested for normality with a Kolmogorov-Smirnov test. For imaging, the statistical significance for multiple comparisons was assessed with 1-way ANOVA with Tukey’s post hoc test. *P* < 0.05 was considered significant.

### Study approval.

The protocols for the human study were approved by the Berkshire Research Ethics Committee (REC 16/SC/0265). The study complied with all relevant ethical regulations for work with human participants and conformed to the Declaration of Helsinki principles and Good Clinical Practice (GCP) guidelines. Written informed consent was received from participants prior to inclusion in the study.

### Data availability.

All data presented in this article are included in the main text and Supporting Data Values file. Any additional requests can be made to the corresponding author.

## Author contributions

EMC and AA performed experiments and contributed to study design, acquisition of data, analysis, and drafting of the manuscript. SB and AO performed experiments and contributed to acquisition of data. USG, RM, SK, FB, SLRJ, PB, AS, and MD contributed to study design, data interpretation, and critical editing of the manuscript. DP contributed to conception and design of the study, data analysis and interpretation, critical revision of the manuscript, and study supervision. The order of first authorship was determined by the relative overall contributions to the study.

## Supplementary Material

Supplemental data

Supplemental video 1

Supplemental video 2

Supporting data values

## Version 1 02/22/2024

Electronic publication
