# Supplementary material for: IL-15 reprogramming compensates for NK cell mitochondrial dysfunction in HIV-1 infection
Source: JCI Insight. 2024 Feb 22;9(4):e173099. doi: 10.1172/jci.insight.173099 (PMC11143930; doi:10.1172/jci.insight.173099)
Supplement: Supplemental data [file jciinsight-9-173099-s078.pdf]

IL-15 re-programming compensates for NK cell mitochondrial dysfunction in HIV-1 infection

Elia Moreno-Cubero[1,9], Aljawharah Alrubayyi[1,9], Stefan Balint[2], Ane Ogbe[1], Upkar S Gill[3], Rebecca Matthews[4], Sabine Kinloch[5], Fiona Burns[4,5], Sarah Rowland-Jones[1], Persephone Borrow[1], Anna Schurich[6], Michael Dustin[2], Dimitra Peppa[5,7, 8*]

Supplementary material:

Supplementary figure 1. SCENITH metabolic analysis of canonical and adaptive NK cells.

Supplementary figure 2. NK cell frequencies after oligomycin treatment.

Supplementary figure 3. Evaluation of NK cell mitochondrial health.

Supplementary figure 4. Quantification of gene expression by RT-qPCR.

Supplementary figure 5. Analysis of NK cell membrane health after IL-15 treatment.

Supplementary movie 1. Representative confocal z-stack, 3D projection and reconstruction of mitochondrial distribution in a primary human NK cell isolated from a HCMV+ control.

Supplementary movie 2. Representative confocal z-stack, 3D projection and reconstruction of mitochondrial distribution in a primary human NK cell isolated from an HIV-1 positive donor.

Supplementary figure 1. SCENITH metabolic analysis of canonical and adaptive NK cells.

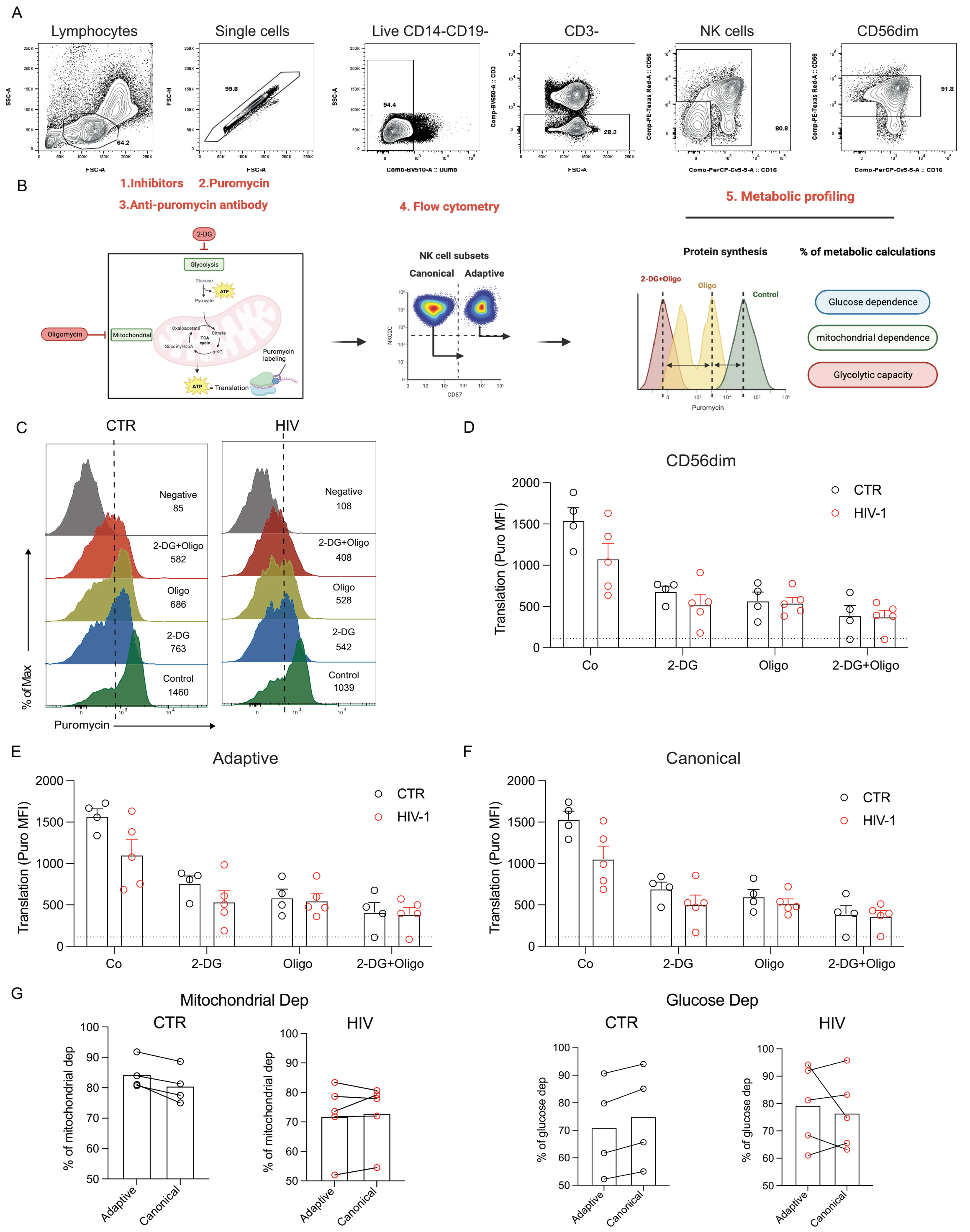

(A) Representative example of flow cytometry plots from a HIV-1 positive subject showing gating on live CD3-, CD14-, CD19- lymphocytes; CD56 and CD16 NK cells. **(B)** Conceptual overview of *ex vivo* SCENITH metabolic profiling of different NK cell subpopulations. **(C)** Representative histogram plots showing levels of puromycin measured by SCENITH in the presence or absence of different inhibitors in NK cells from healthy controls (CTR n=4) and HIV-1 positive donors (HIV-1; n=5). **(D)** Translation levels at basal and after Oligomycin or 2-DG treatments in CD56dim, **(E)** adaptive, and **(F)** canonical NK cell subsets. Doted black line represents the background level obtained after negative control treatment. **(G)** Dependency of adaptive and canonical NK cells on mitochondrial or glucose oxidation in HIV-1 negative and HIV-1 positive donors.

Supplementary figure 2. NK cell frequencies after the oligomycin treatment.

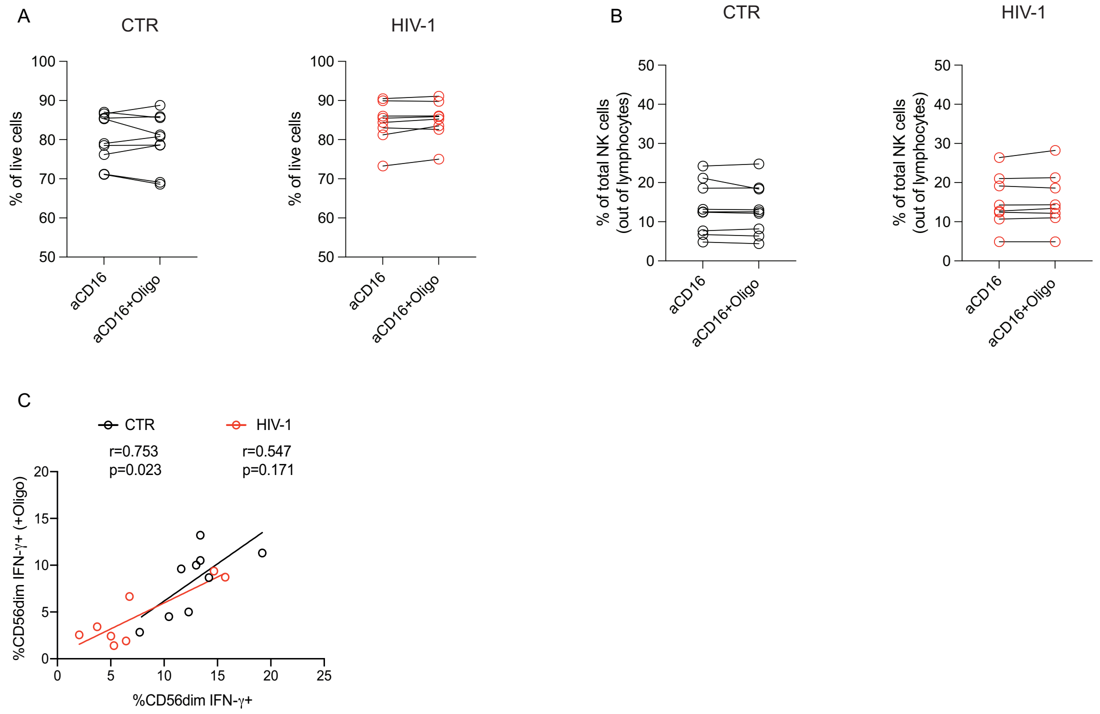

(A) Percentage of live cells and **(B)** total NK cells (out of lymphocytes) before and after the addition of oligomycin. **(C)** Correlation between the percentage of CD56dim IFN-γ+ cells after CD16 stimulation in the presence or absence of oligomycin.

Supplementary figure 3. Evaluation of NK cell mitochondrial health.

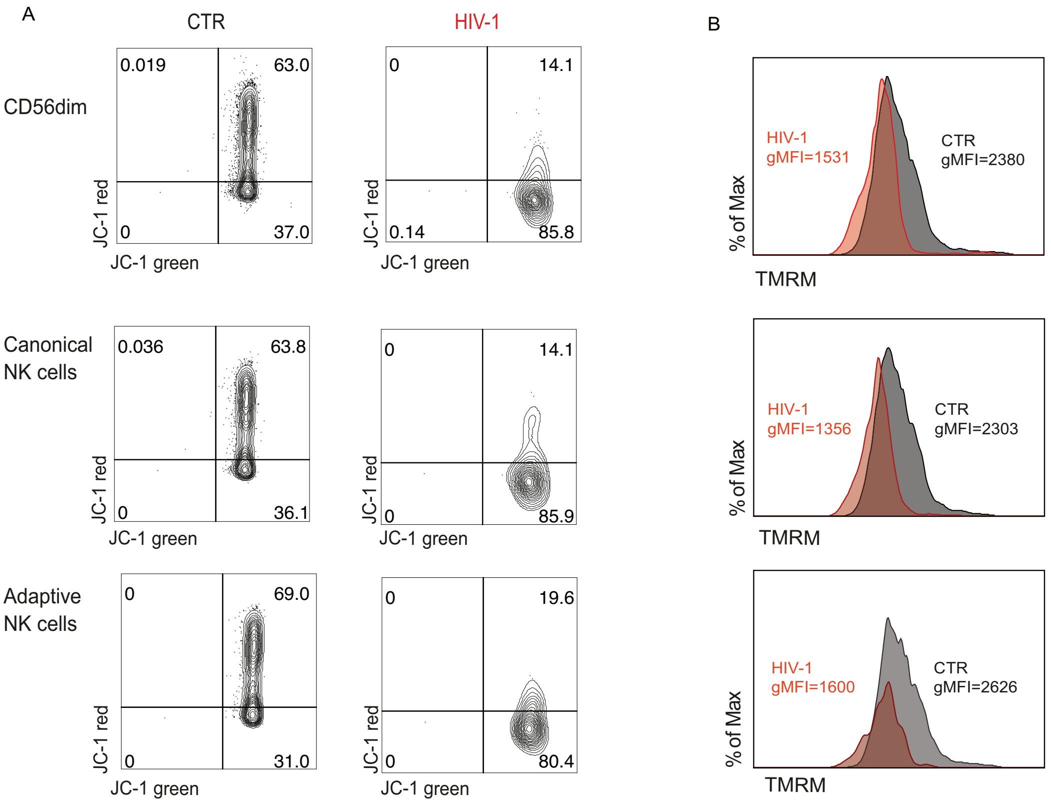

(A) Representative flow plots depicting the ratio of polarised over depolarised mitochondrial of total CD56dim NK cells and NK cell subsets by JC-1 staining in a control (CTR) and HIV-1 positive donor (HIV-1). **(B)** Representative histograms of TMRM gMFI in CD56dim NK cells and NK cell subsets in the same HIV-negative and -positive donors.

Supplementary figure 4. Quantification of gene expression by RT-qPCR.

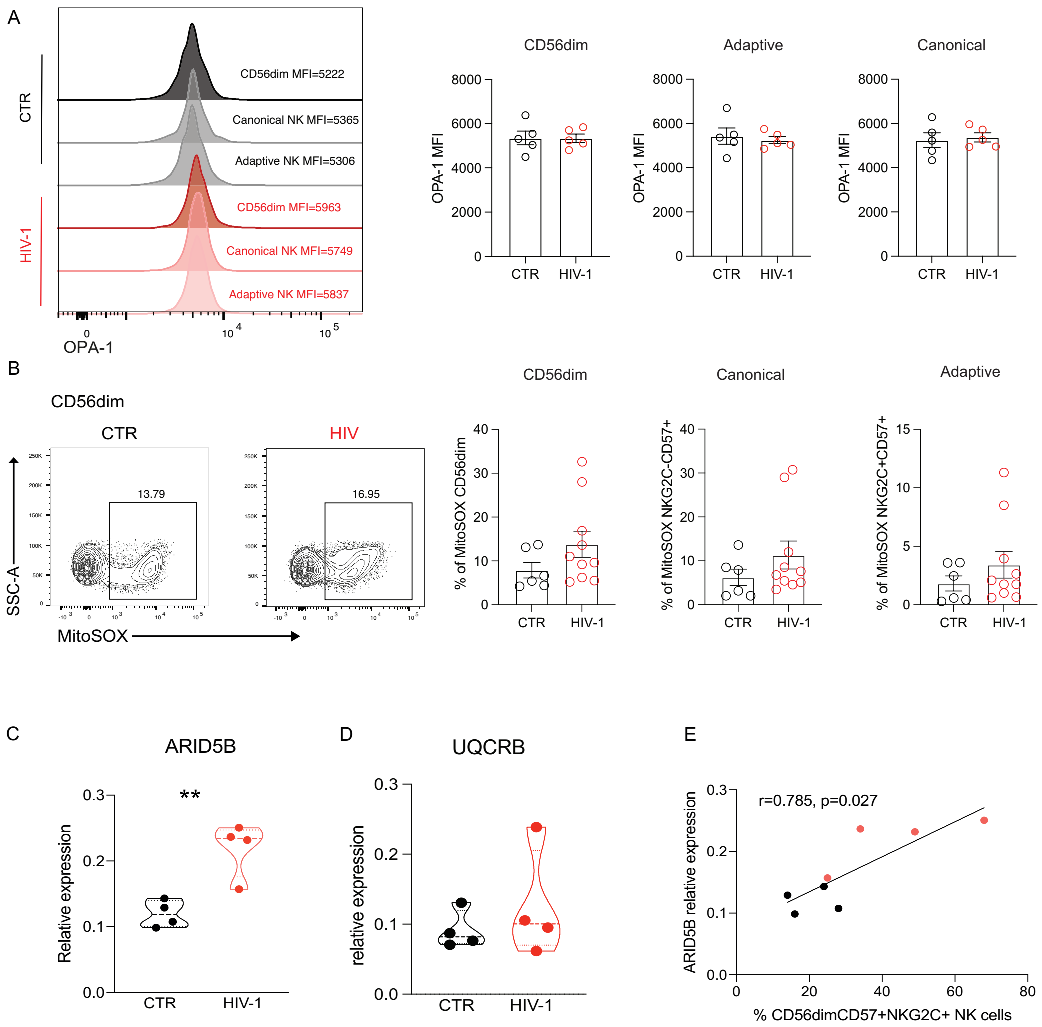

(A) Histogram plots and summary analysis showing the expression levels of OPA-1 in NK cell subsets from healthy controls (CTR) and HIV-1 positive patients (HIV-1). **(B)** The percentage of MitoSOX+ cells in the NK cell subpopulations. **(C)** *ARID5B* and **(D)** *UQCRB* expressions in isolated NK cells from n=4 HCMV+ HIV-negative controls and n=4 HIV-1 positive subjects. All relative expression values were calculated by normalising against GAPDH as a housekeeping gene. **(E)** Correlation between levels of expression of *ARID5B* and *ex vivo* frequencies of adaptive CD57+NKG2C+ NK cells. Significance determined by Mann-Whitney U test. The non-parametric Spearman test was used for correlation analysis. **p<0.01.

Supplementary figure 5. Analysis of NK cell membrane health after IL-15 treatment.

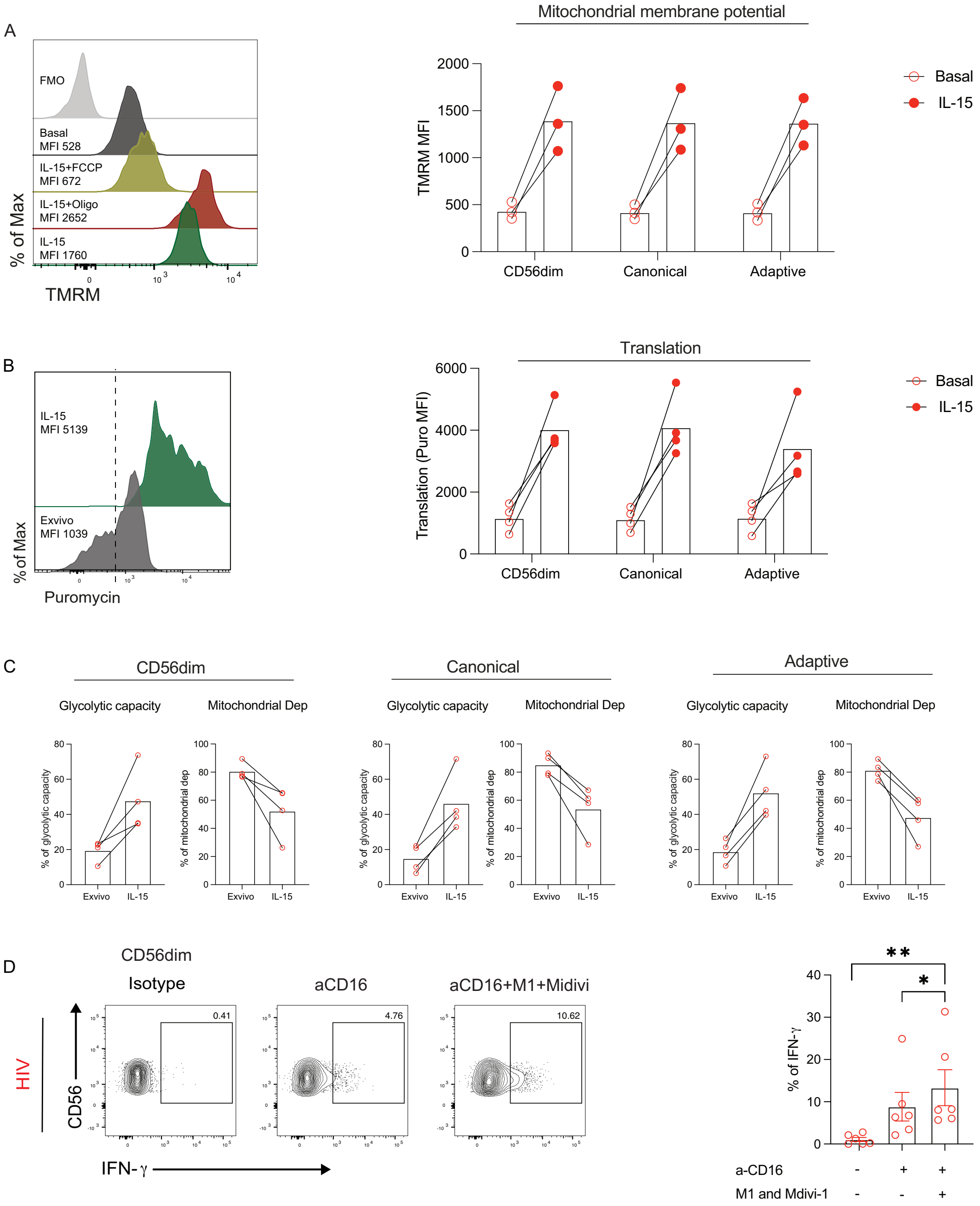

(A) Representative histograms and paired analysis showing TMRM MFI *ex vivo* and following IL-15 treatment in NK cell subsets from n=3 HIV-1 positive donors. FCCP (negative) and Oligomycin (positive) were used as controls for the TMRM measurement. **(B)** Analysis of puromycin levels measured by SCENITH *ex vivo* and following IL-15 treatment in NK cells from n=4 HIV-1 positive individuals. **(C)** Glycolytic capacity and the mitochondrial dependence in NK cell subsets at basal level and following IL-15 treatment. **(D)** IFN-γ production by CD56dim NK cells following anti-CD16 stimulation alone or in the presence of M1 and Mdivi-1 as indicated. Significance determined by one-way ANOVA with multiple comparisons test (D). *p<0.05.

Supplementary movie 1. Representative confocal z-stack, 3D projection and reconstruction of mitochondrial distribution in a primary human NK cell isolated from a CMV+ control. Mitochondria, red; cell membrane, green.

Supplementary movie 2. Representative confocal z-stack, 3D projection and reconstruction of mitochondrial distribution in a primary human NK cell isolated from an HIV-1 seropositive donor. Mitochondria, red; cell membrane, green.
